# Role of the Interleukin 10 Family of Cytokines in Patients With Immune Reconstitution Inflammatory Syndrome Associated With HIV Infection and Tuberculosis

**DOI:** 10.1093/infdis/jit002

**Published:** 2013-01-09

**Authors:** Rebecca Tadokera, Katalin A. Wilkinson, Graeme A. Meintjes, Keira H. Skolimowska, Kerryn Matthews, Ronnett Seldon, Molebogeng X. Rangaka, Gary Maartens, Robert J. Wilkinson

**Affiliations:** 1 Clinical Infectious Diseases Research Initiative, Institute of Infectious Diseases and Molecular Medicine; 2 Division of Clinical Pharmacology; 3 Department of Medicine, University of Cape Town, Cape Town; 4 Infectious Diseases Unit, G. F. Jooste Hospital, Manenberg, South Africa; 5 Department of Medicine, Imperial College London; 6 MRC National Institute for Medical Research, London, United Kingdom

**Keywords:** tuberculosis, HIV-1, complications, immune response, interleukin-10, drug therapy, immune reconstitution inflammatory syndrome, interleukin-22, interleukin-26

## Abstract

***Background.***  The interleukin 10 (IL-10) family comprises cytokines structurally related to IL-10 that share signaling receptors that have conserved signaling cascades. The immunopathogenesis of immune reconstitution inflammatory syndrome (IRIS) in patients with human immunodeficiency virus (HIV) infection and tuberculosis remains incompletely understood. We hypothesized that a deficiency of IL-10 and its homologs may contribute to the immunopathology of IRIS in these patients.

***Methods.*** We performed a case-control analysis involving patients with HIV infection and tuberculosis who had IRIS at clinical presentation (tuberculosis-IRIS) and similar patients with HIV infection and tuberculosis who did not develop tuberculosis-IRIS (non-IRIS). Peripheral blood mononuclear cells (PBMCs) were cultured in the presence or absence of heat-killed *Mycobacterium tuberculosis* for 6 and 24 hours. Messenger RNA was analyzed by quantitative reverse transcription polymerase chain reaction analysis. Cytokine concentrations in serum were also determined.

***Results.*** Cultures of PBMCs stimulated with *M. tuberculosis* for 24 hours yielded higher IL-10 and interleukin 22 (IL-22) transcript levels for tuberculosis-IRIS patients, compared with non-IRIS patients. Analysis of corresponding serum samples showed significantly higher concentrations of IL-10 and IL-22 in tuberculosis-IRIS patients, compared with non-IRIS patients.

***Conclusions.*** IL-10 and IL-22 were differentially induced in PBMCs from tuberculosis-IRIS patients after in vitro stimulation, and higher concentrations of their corresponding proteins were detected in serum (in vivo). The higher levels of IL-10 observed in this study may represent a compensatory antiinflammatory response during tuberculosis-IRIS. The elevated levels of IL-22 suggest an association between this cytokine and immunopathology during tuberculosis-IRIS.

The immune reconstitution inflammatory syndrome (IRIS) is a commonly encountered immunological complication of the combined therapies for human immunodeficiency virus type 1 (HIV-1) infection and tuberculosis [1, 2]. Two forms of tuberculosis-associated IRIS (tuberculosis-IRIS) exist. The condition is best recognized as a paradoxical immune-mediated worsening of tuberculosis symptoms and other symptoms occurring in patients prescribed combined antitubercular and antiretroviral therapy [3, 4]. Common clinical manifestations of tuberculosis-IRIS include recurrent tuberculosis symptoms, fever, and enlarging and suppuration of lymph nodes [4]. Understanding of the immunopathogenesis of tuberculosis-IRIS remains incomplete, although it has become the subject of increased investigation in recent years.

We previously investigated the involvement of multiple cytokines and demonstrated that paradoxical tuberculosis-IRIS is associated with hypercytokinemia [5, 6]. The interleukin 10 (IL-10) family comprises cytokines structurally related to IL-10, such as interleukin 19 (IL-19), interleukin 20 (IL-20), interleukin 22 (IL-22), interleukin 24 (IL-24; MDA-7), interleukin 26 (IL-26; AK155), interleukin 28 (IL-28), and interleukin 29 (IL-29) in humans, in addition to some encoded in the genomes of herpes and pox viruses [7]. IL-10–related cytokines are highly pleiotropic but are linked together through genetic similarity and intron-exon gene structure [8], although other than the activities of IL-10, little is known about the biological activities of the IL-10 family [7]. It has been suggested that these cytokines may participate in T-cell–mediated diseases by regulation of T-cell cytokine profiles [9]. Human IL-10 is the most well-characterized molecule in this group, and it has multiple biological effects, including immunoregulatory and antiinflammatory effects on different cell types. IL-10 is produced by various immune cells, which include activated monocytes, T cells (CD4^+^ and CD8^+^), macrophages, and dendritic cells [10]. IL-10 functions mainly to regulate T-helper 1 cytokines, major histocompatibility class II and B7 molecules, and expression of costimulator molecules on macrophages that are necessary for optimal T-cell activation [7, 8, 11]. By inhibiting expression of these molecules on antigen-presenting cells, IL-10 directly suppresses the activation of T cells and the production of T-cell–derived cytokines, such as interferon γ (IFN-γ) and interleukin 2 [11].

Similar to IL-10, the related members of the IL-10 family are secreted as α helical proteins whose amino acid sequences are up to 30% identical to that of IL-10, with the encoding genes located on 2 clusters in the human genome [12, 13]. One major similarity among these cytokines is the sharing of signaling receptors and the use of conserved signaling cascades [8]. However, knowledge of the biology of many of these IL-10 homologs remains incomplete. IL-22 is a proinflammatory cytokine that plays an important role in innate pathogen defense [12,14]. Expression of IL-22 has been shown to be highest in activated memory CD4^+^ T cells and lowest in activated natural killer cells [8], with little or no expression observed in other immune cells [13]. The preferential production of IL-22 by T cells suggests that increased expression of this cytokine may occur in T-cell–mediated diseases [8]. IL-22 acts on nonimmune cells and has been linked with severe inflammation in chronic T-cell–mediated inflammatory diseases, such as psoriasis, Crohn disease, and rheumatoid arthritis [12, 13]. IL-22 also contributes to the human antimycobacterial immune response [15, 16].

The resolution of inflammation is dependent on the host's ability to mount an immunoregulatory response to regulate the magnitude of inflammatory responses. IRIS results from excessive pathological immune responses occurring during immune recovery in HIV-infected patients who are commencing antiretroviral therapy (ART). In this study, we therefore hypothesized that IL-10 and its homologs may be deficient and that this immune dysregulation may be involved in the immunopathology associated with tuberculosis-IRIS.

## METHODS

Ethical approval for these studies was provided by the University of Cape Town Research Ethics committee (REC references 337/2004 and 173/2005). Participants were recruited at G. F. Jooste Hospital and at the Ubuntu Clinic, Site B Khayelitsha, in Cape Town, South Africa, between March 2005 and December 2007 to 2 prospective studies whose designs have been previously reported [17, 18]. Patients had a confirmed diagnosis of paradoxical tuberculosis-IRIS that was based on the International Network for the Study of HIV-associated IRIS consensus case definition [3], which has been independently validated [19–21]. Patients were >18 years of age, not pregnant, and ART naive. Known rifampicin-resistant tuberculosis was an exclusion criterion. At entry into these studies, a clinical diagnostic work-up was performed to exclude an alternative diagnosis for clinical deterioration.

### Cross-sectional Analysis of Paradoxical Tuberculosis-IRIS and Non-IRIS Patients

For the case-control analysis, 20 patients (of 32 available) with paradoxical tuberculosis-IRIS were selected randomly from participants who were enrolled at the time of diagnosis with paradoxical tuberculosis-IRIS. Cases were matched with 20 non-IRIS controls who were enrolled using similar criteria (CD4^+^ T-cell count, age, sex, and duration of anti-tuberculosis treatment) but did not develop tuberculosis-IRIS 2 weeks after starting combination ART (cART). The study design, patient selection criteria, and treatment regimens have been previously described [5].

### Blood Processing and Cell Cultures

Peripheral blood mononuclear cells (PBMCs) were isolated using Ficoll from 30 mL of blood collected in Na-heparin BD vacutainers and were plated at 2.5 × 10^6^ cells/well. PBMCs were restimulated with heat-killed H37Rv *Mycobacterium tuberculosis* for 6 or 24 hours at a multiplicity of infection (MOI) of 1:1. After incubation, PBMCs were harvested and lysed for RNA extraction. Cell lysates from both the 6- and 24-hour time points were stored for RNA analysis. Serum was also collected and cryopreserved at −80°C until further analysis.

### Quantitative Reverse Transcription Polymerase Chain Reaction (qRT-PCR)

Messenger RNA (mRNA) was extracted from PBMC lysates by using the RNeasy Mini Kit Spin Protocol (Qiagen, Valencia, CA) in accordance with the manufacturer's recommendations and was cryopreserved at −80°C until further analysis. Primers and probes were purchased from Applied Biosystems as inventoried TaqMan Gene Expression Assays (Applied Biosystems, Qiagen, Valencia, CA). The TaqMan RNA-Ct 1 Step kit protocol was used in these assays. A lower difference in cycle threshold (ΔCt) indicates a higher transcript abundance. Fold-induction was used to obtain a relative measure of gene induction by *M. tuberculosis* and was determined using the ΔΔCt method.

### Analysis of Cytokine Levels in Serum Samples

The level of soluble IL-10 protein in serum samples was measured using customized commercial Milliplex XMAP kits, whereas the level of IL-22 was measured using the human IL-22 Immunoassay Quantikine ELISA kit from R&D Systems (Minneapolis, MN).

### Flow Cytometric Analysis

Frozen PBMCs from 4 patients with tuberculosis-IRIS were thawed, resuspended in Roswell Park Memorial Institute medium (Sigma, UK) with 10% heat-inactivated fetal calf serum (FCS; Sigma, UK), and stimulated with heat-killed *M. tuberculosis* H37Rv (hkH37Rv; MOI 1:1) for 4 hours at 37°C in 5% CO_2_. Brefeldin A (10 μg/mL; Sigma, UK) was added to each tube, vortexed, and incubated for a further 20 hours. After 24 hours, cells were washed twice with phosphate-buffered saline (PBS; Sigma, UK) and stained for surface CD3-FITC and CD14-APC (both from BD Biosciences) for 30 minutes in the dark at 4°C. PBMCs were washed in fluorescence-activated cell sorter (FACS) wash buffer (PBS with 0.5% FCS); BD Cytofix/Cytoperm was then added, and PBMCs were incubated for 20 minutes in the dark at 4°C. PBMCs were washed with 1X BD Perm Wash and stained for intracellular IL-10-PE (BD Biosciences) and IL-22-PerCP-eFluor 710 (eBiosciences) for 1 hour at 4°C in the dark. Cells were washed 3 times with 1X BD Perm Wash buffer and acquired on a BD FACS Calibur. Analysis was performed using FlowJo, v9.4.3 (available at: http://www.treestar.com).

## RESULTS

### Baseline Characteristics of Participants

A total of 20 patients with paradoxical tuberculosis-IRIS and 20 non-IRIS controls were analyzed. Supplementary Table 1 shows a summary of the baseline demographic and clinical characteristics for the patients analyzed in this study. The 2 groups of patients were well matched with respect to sex, age, and baseline CD4^+^ T-cell count. There were no significant differences between the tuberculosis-IRIS and non-IRIS groups in terms of previous tuberculosis, tuberculosis form, and median days from cART initiation to IRIS onset (or to sample collection, in the case of non-IRIS controls). However, tuberculosis-IRIS patients were more likely to have a shorter duration between tuberculosis treatment and commencement of cART (*P* = .028).

### Transcript Abundance of IL-10–Related Genes

PBMCs from 20 tuberculosis-IRIS patients and 20 non-IRIS controls were cultured in the presence or absence of heat-killed H37Rv *M. tuberculosis* antigen for 6 and 24 hours. Evaluation at 6 hours revealed that *M. tuberculosis* stimulation had increased the transcript abundance of several of the cytokines in both the tuberculosis-IRIS and non-IRIS groups (Table 1). A lower ΔCt indicates a higher transcript abundance. Significantly higher transcript levels were observed for IL-22 in tuberculosis-IRIS patients after stimulation (*P* = .009), whereas levels of IL-24 transcripts were higher for non-IRIS patients (*P* = .020). IL-10 levels were significantly higher in unstimulated non-IRIS cultures (*P* = .04) and increased more in tuberculosis-IRIS cultures, compared with non-IRIS cultures, after stimulation. IL-26 transcript levels were significantly higher in both stimulated and unstimulated cultures of tuberculosis-IRIS PBMCs (*P* = .008 and *P* = .042, respectively). IL-28 transcript levels did not differ between unstimulated tuberculosis-IRIS and non-IRIS cultures but increased significantly in non-IRIS cultures after stimulation (*P* = .013).

**Table 1.** Cycle Threshold Differences (ΔCt) for Interleukin 10 (IL-10)–Related Cytokine Genes After 6 Hours of In Vitro Stimulation With Heat-Killed *Mycobacterium tuberculosis*

Cytokine					*P*			
	Unstimulated PBMCs, by Study Group, Median (IQR)		Stimulated PBMCs, by Study Group, Median (IQR)		Tuberculosis-IRIS vs Non-IRIS		Unstimulated vs Stimulated	
	Tuberculosis-IRIS	Non-IRIS	Tuberculosis-IRIS	Non-IRIS	Unstimulated	Stimulated	Tuberculosis-IRIS	Non-IRIS
IL-10	7.2 (6.4–8.2)	6.6 (5.8–7.6)	5.9 (3.4–7.1)	6.1 (5.0–7.5)	.04	.25	.003	.27
IL-19	13.4 (5.5–17.8)	13.6 (10.8–19.2)	8.8 (6.5–11.6)	8.4 (5.1–12.1)	.935	.804	<.0001	<.0001
IL-20	18.9 (11.2–21.3)	18.4 (9.6–21.2)	14.3 (10.6–19.8)	13.2 (8.5–18.8)	.236	.103	<.0001	<.0001
IL-22	19.5 (15.6–21.6)	18.6 (13.8–21.3)	13.0 (8.3–20.5)	17.1 (10.1–21.4)	.073	.009	<.0001	.086
IL-24	14.1 (7.6–20.8)	13.3 (5.8–18.3)	11.8 (7.7–15.7)	9.5 (5.9–13.7)	.625	.020	.001	<.0001
IL-26	16.9 (14.4–20.0)	18.6 (17.1–21.0)	16.4 (11.3–19.4)	17.5 (13.0–20.7)	.008	.042	.002	.010
IL-28	11.6 (8.6–16.3)	11.7 (9.7–13.0)	12.5 (10.1–14.3)	11.4 (8.8–12.8)	.882	.013	.351	.130

Data are for patients with HIV infection and tuberculosis who had IRIS at clinical presentation (tuberculosis-IRIS) and similar patients with HIV infection and tuberculosis who did not develop tuberculosis-IRIS (non-IRIS). At 6 hours, levels of IL-22 transcripts in stimulated PBMC cultures were significantly higher in tuberculosis-IRIS patients, whereas levels of IL-24 transcripts stimulated cultures were higher in non-IRIS patients. IL-26 had significantly higher transcript levels in both stimulated and unstimulated PBMC cultures for tuberculosis-IRIS patients. Levels of IL-28 transcripts were marginally higher in the stimulated PBMC cultures for non-IRIS patients (*P* = .013). IL-29 transcripts were barely detectable (results not shown). A lower ΔCt indicates a higher transcript abundance.

Abbreviations: HIV, human immunodeficiency virus; IL-19, interleukin 19; IL-20, interleukin 20; IL-22, interleukin 22; IL-24, interleukin 24; IL-26, interleukin 26; IL-28, interleukin 28; IQR, interquartile range; IRIS, immune reconstitution inflammatory syndrome; PBMC, peripheral blood mononuclear cell.

At 24 hours, several of the cytokines (IL-10, IL-20, and IL-26) had significantly higher transcript levels in the stimulated tuberculosis-IRIS cultures (*P* < .001, *P* = .022, and *P* = .004, respectively). IL-22 transcript levels were higher in both stimulated and unstimulated tuberculosis-IRIS cultures (*P* = .004 and *P* = .015, respectively). IL-28 transcript levels were significantly higher in unstimulated non-IRIS PBMCs (*P* = .012) but not in stimulated cultures. In PBMCs from both tuberculosis-IRIS and non-IRIS patients, *M. tuberculosis* stimulation resulted in increased transcript abundance for many of the cytokines, most prominently IL-19, IL-20, IL-24, and IL-26 (Table 2).

**Table 2.** Cycle Threshold Differences (ΔCt) for Interleukin 10 (IL-10)–Related Cytokine Genes After 24 Hours of In Vitro Stimulation With Heat-Killed *Mycobacterium tuberculosis*

Cytokine					*P*			
	Unstimulated PBMCs, by Study Group, Median (IQR)		Stimulated PBMCs, by Study Group, Median (IQR)		Tuberculosis-IRIS vs Non-IRIS		Unstimulated vs Stimulated	
	Tuberculosis-IRIS	Non-IRIS	Tuberculosis-IRIS	Non-IRIS	Unstimulated	Stimulated	Tuberculosis-IRIS	Non-IRIS
IL-10	6.9 (5.1–8.1)	6.9 (6.0–7.1)	4.9 (3.1–6.4)	6.8 (5.9–8.5)	.74	<.001	<.001	1.0
IL-19	11.7 (2.3–16.9)	10.9 (4.3–18.8)	6.9 (2.9–10.1)	5.9 (2.0–18.2)	.472	.433	<.0001	.0002
IL-20	14.0 (7.9–19.1)	16.2 (4.3–20.8)	9.6 (5.9–15.9)	12.5 (6.4–19.1)	.386	.028	.0003	<.0001
IL-22	18.3 (12.4–19.9)	15.5 (12.5–19.7)	12.0 (6.8–14.9)	14.7 (8.0–19.2)	.004	.015	<.0001	.199
IL-24	13.2 (6.4–18.3)	13.1 (3.1–14.0)	10.4 (5.5–15.1)	9.9 (12.9–15.1)	.944	.661	.007	.020
IL-26	13.1 (10.2–16.5)	13.3 (11.4–18.2)	8.4 (4.4–14.5)	11.1 (7.2–16.2)	.273	.004	<.0001	.019
IL-28	10.7 (8.6–14.2)	9.7 (7.5–11.8)	10.2 (6.4–12.3)	10.2 (8.3–11.9)	.012	.609	.034	.025

Data are for patients with HIV infection and tuberculosis who had IRIS at clinical presentation (tuberculosis-IRIS) and similar patients with HIV infection and tuberculosis who did not develop tuberculosis-IRIS (non-IRIS). At 24 hours, IL-10, IL-20, and IL-26 transcript levels were significantly higher in the stimulated cultures for tuberculosis-IRIS patients but not in unstimulated cultures. IL-22 transcript levels were higher in unstimulated non-IRIS cultures but increased significantly after *M. tuberculosis* stimulation in tuberculosis-IRIS patients only (*P* = .015). IL-28 transcript levels were significantly higher in non-IRIS patients at 24 hours in unstimulated cultures but not in stimulated cultures. A lower ΔCt indicates a higher transcript abundance.

Abbreviations: HIV, human immunodeficiency virus; IL-19, interleukin 19; IL-20, interleukin 20; IL-22, interleukin 22; IL-24, interleukin 24; IL-26, interleukin 26; IL-28, interleukin 28; IQR, interquartile range; IRIS, immune reconstitution inflammatory syndrome; PBMC, peripheral blood mononuclear cell.

### Fold-Induction Analysis of IL-10–Related Genes

**Figure 1.**
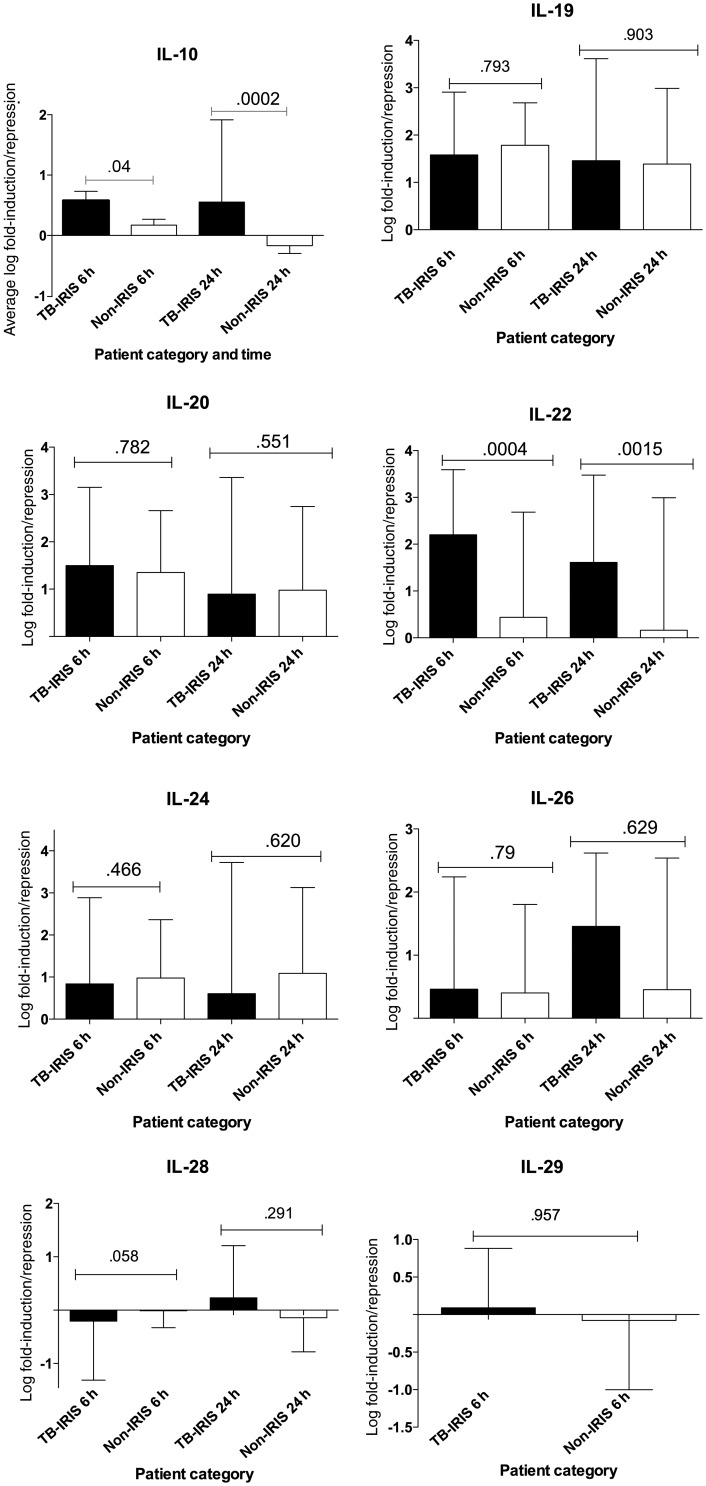
Fold-induction analysis of induction of interleukin 10 (IL-10)–related cytokines by heat-killed *Mycobacterium tuberculosis*. Peripheral blood mononuclear cells (PBMCs) from 20 patients with human immunodeficiency virus (HIV) infection and tuberculosis who had immune reconstitution inflammatory syndrome (IRIS) at clinical presentation (tuberculosis-IRIS) and 20 control patients with HIV infection and tuberculosis who did not develop tuberculosis-IRIS (non-IRIS) were cultured in the presence or absence of *M. tuberculosis* for 6 and 24 hours and then lysed for messenger RNA transcript analysis by quantitative reverse transcription polymerase chain reaction. Fold-induction of the genes was determined using the ΔΔ cycle threshold method. This method compares the difference in the cycle threshold between the gene of interest to that of a normalization gene (β-actin). IL-10 and interleukin 22 (IL-22) were differentially induced in tuberculosis-IRIS patients at both 6 and 24 hours. For interleukin 19 (IL-19), interleukin 20 (IL-20), interleukin 24 (IL-24), interleukin 26 (IL-26), interleukin 28 (IL-28), and interleukin 29 (IL-29), no significant differences were noted between tuberculosis-IRIS and non-IRIS patients, irrespective of restimulation of PBMCs with *M. tuberculosis*. Tuberculosis-IRIS patients are represented by the black boxes, whereas the open white boxes represent non-IRIS patients.

IL-10 mRNA was highly and significantly induced in tuberculosis-IRIS patients at 6 hours and more so at 24 hours (*P* = .04 and *P* = .0002, respectively; Figure 1). Similarly, IL-22 was found to be significantly upregulated at both 6 and 24 hours in cultures from tuberculosis-IRIS patients, compared with non-IRIS control patients (*P* = .004 and *P* = .0015, respectively). Although both IL-19 and IL-20 were highly induced by *M. tuberculosis* stimulation, no significant differences were observed when comparing mRNA from tuberculosis-IRIS patients and mRNA from non-IRIS patients in cultures from the 6- and 24-hour time points. Although IL-24 tended to be more induced in cultures for the non-IRIS group at both time points, the difference between the tuberculosis-IRIS and non-IRIS groups was not significant. Similarly, there were no significant differences observed between tuberculosis-IRIS and non-IRIS patients at either time point for IL-26, although there tended to be more induction of IL-26 in the tuberculosis-IRIS patients at 24 hours. IL-28 and IL-29 were seldom expressed at high levels in these samples, and no significant differences were observed between induction/repression in the 2 patient groups. Although IL-29 was marginally detectable at 6 hours, it was not detectable at 24 hours (Figure 1).

### Analysis of Serum Cytokine Levels

**Figure 2.**
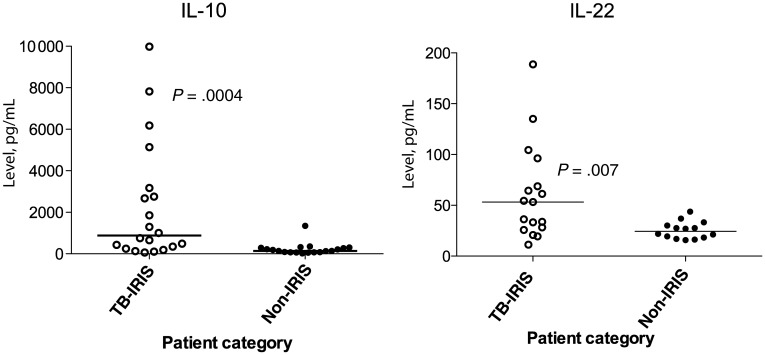
Analysis of interleukin 10 (IL-10) and interleukin 22 (IL-22) concentrations in serum. Consistent with the fold-induction analysis, IL-10 and IL-22 protein levels were differentially higher in the serum of patients with human immunodeficiency virus (HIV) infection and tuberculosis who had immune reconstitution inflammatory syndrome (IRIS) at clinical presentation (tuberculosis-IRIS). Tuberculosis-IRIS patients are shown by the white circles, whereas the open black circles represent the control patients with HIV infection and tuberculosis who did not develop tuberculosis-IRIS (non-IRIS).

To further investigate the RNA results, protein concentrations were determined in serum samples for genes that showed consistent and significant differences in the mRNA analysis (ie, IL-10 and IL-22). Significantly higher concentrations of IL-10 (*P* = .0004; Figure 2) were detected in the serum of tuberculosis-IRIS patients (median, 875.6 pg/mL [interquartile range {IQR}, 64.5–9982 pg/mL]), compared with non-IRIS patients (median, 137.2 pg/mL [IQR, 24.3–1346 pg/mL]). Similarly, significantly higher levels of IL-22 (*P* = .007) were detected in the serum samples of tuberculosis-IRIS patients (median, 53.2 pg/mL [IQR, 11.31–188.8 pg/mL]), compared with non-IRIS patients (median, 24.4 pg/mL [IQR, 15.7–43.8 pg/mL]).

### Correlation of IL-10 and IL-22 Serum Concentrations

To determine whether the elevated concentrations of IL-10 and IL-22 observed in the serum samples of tuberculosis-IRIS patients correlated, Spearman correlation was calculated. There was an inverse correlation between the serum concentrations of IL-10 and IL-22 (Spearman r = −0.69; *P* = .007) in the tuberculosis-IRIS group. However, no significant correlation was observed in the non-IRIS group (Spearman r = −0.07; *P* = .83).

### Cellular Origin of IL-10 and IL-22

To investigate the cellular origin of IL-10 and IL-22 in tuberculosis-IRIS patients, frozen PBMCs from 4 patients who presented with IRIS were stimulated with heat-killed *M. tuberculosis* H37Rv (MOI 1:1) for 4 hours, followed by addition of brefeldin A for a further 20 hours. At this time, cells were stained for surface CD3 and CD14. Cells were then stained for intracellular IL-10 and IL-22. We found that CD14^+^ cells were more likely to be positive for IL-22 (median, 0.6% [IQR, 0.02%–2.1%]), compared with CD3^+^ lymphocytes (median, 0.02% [IQR, 0%–0.04%]). The difference was not statistically significant, owing to the small number of samples. Similarly, CD14^+^ cells were also more likely to be positive for IL-10 (median, 0.06% [IQR, 0%–0.15%), compared with CD3^+^ cells (median, 0% [IQR, 0%–0.01%]).

## DISCUSSION

Our analysis of IL-10–related cytokines showed an increase in the transcript levels of IL-10 and IL-22 in tuberculosis-IRIS patients, compared with non-IRIS controls. The corresponding serum samples showed significantly higher concentrations of IL-10 and IL-22 protein in tuberculosis-IRIS patients. Thus, our hypothesis that IRIS is associated with depressed IL-10 responses was falsified, as the opposite appears to have been the case. Whereas analysis of fold-induction showed significant induction of IL-19, IL-20, IL-24, and IL-26 by *M. tuberculosis*, the differences detected between the tuberculosis-IRIS and non-IRIS groups were neither great nor statistically significant.

Whereas the regulatory role of IL-10 in vitro and in animal models is well documented, the role of this cytokine in clinical situations, particularly in infectious diseases, remains the subject of investigation [9]. Production of IL-10 can also be stimulated by bacteria, viruses, and parasites and is regulated both at the transcriptional and translation levels. The absence of IL-10 in knockout mice results in reduced *M. tuberculosis* loads in the lung. However, this reduction was preceded by an accelerated and enhanced IFN-γ response in the lung, an increased influx of CD4^+^ T cells into the lung, and enhanced production of chemokines and cytokines, including CXCL10 and IL-17, in both the lung and the serum [22]. This suggests that, although the early production of IL-10 in response to tuberculosis may be detrimental, this cytokine is ultimately necessary to prevent immunopathology.

IL-10 has been reported to modulate the innate and adaptive immune responses, potentially creating a favorable environment for the persistence of microbes, intracellular pathogens, and chronic infections [9]. The increased ability of macrophages to produce IL-10 when stimulated with Toll-like receptor ligands is also associated with an increased tendency to develop primary progressive tuberculosis [22]. Production of IL-10 has also been reported to be higher in patients who had active tuberculosis, compared with tuberculin skin test responders [23]. In this study, we show that consistently higher levels of IL-10 mRNA and serum protein were observed in tuberculosis-IRIS patients. Tuberculosis-IRIS is a highly inflammatory condition. We hypothesize that the high levels of IL-10 observed in the peripheral blood of tuberculosis-IRIS patients in this study reflect an overspill of IL-10 from the sites of inflammation where IL-10 would be involved in regulating and resolving inflammation.

Other human studies have shown that IL-10 may be associated with susceptibility to infections caused by rapidly growing mycobacteria [24]. Significantly higher levels of circulating IL-10 have been demonstrated in HIV-infected patients than in healthy controls [25]. IL-10 production has previously been shown to increase in *Mycobacterium avium–*stimulated monocytes from HIV-infected patients, with the highest expression observed in patients with advanced AIDS [24].

It is interesting that levels of IL-22 gene expression and serum protein were, like those of IL-10, found to be significantly higher in tuberculosis-IRIS patients. IL-22 is implicated in T-lymphocyte disease, innate pathogen defense, and acute phase responses [9] and has been associated with increased innate immunity in tissues [13], with expression being higher in outer body barriers, such as skin, the respiratory system, and the digestive system. Previous work on IL-22 and IL-17 in tuberculosis immunity has demonstrated elevated levels of IL-22 in the bronchoalveolar lavage fluid of pulmonary tuberculosis patients [15]. We show that IL-22 may be implicated not only in tuberculosis pathology, but also in tuberculosis-IRIS immunopathology, as indicated by the higher concentrations of IL-22 we observed in tuberculosis-IRIS patients. The coexpression of IL-10 and IL-22 seen in our study and their inverse correlation in tuberculosis-IRIS patients supports the suggestion that there may be interaction between these cytokines in this condition (and not in controls), as has been postulated for other diseases [26]. We also performed preliminary analysis of the cellular source of IL-10 and IL-22 and found both more frequently in CD14^+^ cells (Table 3), consistent with increasing evidence that innate immune system activation is a component of the immunopathology of tuberculosis-IRIS [5, 27–30].

**Table 3.** Cellular Sources of Interleukin 10 (IL-10) and Interleukin 22 (IL-22)

Cells (n = 4)	IL-10		IL-22	
	Unstimulated	hkH37Rv (1:1)	Unstimulated	hkH37Rv (1:1)
CD14^+^	0 (0–0.6)	0.06 (0–0.15)	0 (0–0.01)	0.6 (0.02–2.1)
CD3^+^	0 (0–0.01)	0 (0–0.01)	0.01 (0–0.03)	0.02 (0–0.04)

Data are median % (interquartile range) of positive cells.

Abbreviation: hkH37Rv, heat-killed *Mycobacterium tuberculosis* H37Rv.

IL-19, like IL-20, has been shown to be produced under inflammatory conditions and is thought to play an important role in the pathogenesis of some inflammatory diseases, such as psoriasis. It has been suggested that these cytokines may be important in autoimmune diseases and may enhance antibacterial and antiviral immunity [8]. However, from our study, we did not show a link between IL-19 or IL-20 with tuberculosis-IRIS immunopathology.

A major strength of our study was close matching of patients with paradoxical tuberculosis-IRIS and controls who did not develop the condition. Apart from the longer duration between initiating tuberculosis treatment and ART in the non-IRIS group, the groups were well matched. We acknowledge some limitations to our study, an important one of which is the insufficiency of 24-hour tissue culture supernatants from these patients. This may have limited our ability to validate and confirm the mRNA transcript findings.

In summary, our findings show elevated levels of IL-10 and IL-22 in patients with tuberculosis-IRIS. The higher levels of IL-10 may represent compensatory antiinflammatory and immune-regulatory responses, whereas elevated IL-22 levels suggest an association with immunopathology in tuberculosis-IRIS.

## Supplementary Data

Supplementary materials are available at *The Journal of Infectious Diseases* online (http://jid.oxfordjournals.org/). Supplementary materials consist of data provided by the author that are published to benefit the reader. The posted materials are not copyedited. The contents of all supplementary data are the sole responsibility of the authors. Questions or messages regarding errors should be addressed to the author.

Supplementary Data
