# Supplementary material for: Role of the Interleukin 10 Family of Cytokines in Patients With Immune Reconstitution Inflammatory Syndrome Associated With HIV Infection and Tuberculosis
Source: J Infect Dis. 2013 Jan 9;207(7):1148–56. doi: 10.1093/infdis/jit002 (PMC3583273; doi:10.1093/infdis/jit002)
Supplement: Supplementary Data [file supp_207_7_1148__index.html]

The role of interleukin-10 family of cytokines in HIV-tuberculosis associated immune reconstitution inflammatory syndrome — Role of the Interleukin 10 Family of Cytokines in Patients With Immune Reconstitution Inflammatory Syndrome Associated With HIV Infection and Tuberculosis — Role of the Interleukin 10 Family of Cytokines in Patients With Immune Reconstitution Inflammatory Syndrome Associated With HIV Infection and Tuberculosis — Supplementary Data 

# Role of the Interleukin 10 Family of Cytokines in Patients With Immune Reconstitution Inflammatory Syndrome Associated With HIV Infection and Tuberculosis

## Supplementary Data

Supplementary Data

**Files in this Data Supplement:**

- Supplementary Data - Docx file
