# Supplementary material for: Role of the Interleukin 10 Family of Cytokines in Patients With Immune Reconstitution Inflammatory Syndrome Associated With HIV Infection and Tuberculosis
Source: J Infect Dis. 2013 Jan 9;207(7):1148–56. doi: 10.1093/infdis/jit002 (PMC3583273; doi:10.1093/infdis/jit002)
Supplement: Supplementary Data [file supp_jit002_jit002supp.docx]

**Supplementary Table Baseline characteristics of 20 TB-IRIS versus 20 non-IRIS patients**

|  | TB-IRIS | non-IRIS | p-value |
| --- | --- | --- | --- |
| n | 20 | 20 | N/A |
| Female n, (%) | 11 (55) | 12 (60) | 0.27 |
| Median age (years, IQR) | 31.4  (23.2-45.7) | 35.8  (22.2- 54.1) | 0.273 |
| Baseline CD4 count | 56  (14.0- 193.0) | 49.5  (5.0- 302) | 0.59 |
| Median days of TB-treatment  Prior to ART (IQR) | 51.5  (14.0- 207.0) | 78.0  (29.0- 173.0) | 0.028 |
| Median days of ART to IRIS onset  or sample (IQR) | 14  (5.0-78.0) | 14  (14-14) | 0.94 |
| Previous TB n (%) | 5 (25) | 4 (20) | 1.00 |
| TB Disease form n (%) |  |  |  |
| Pulmonary TB only | 10 (50) | 16 (80) | 0.096 |
| Disseminated TB | 10 (50) | 4 (20) | 0.096 |
| Smear and culture positive n, (%) | 10 (50) | 13 (65) | 0.52 |

20 IRIS and 20 non-IRIS patients were analysed in this study. The were no differences between the two patient groups in terms of previous TB disease, TB disease form and median days of cART to IRIS. However, TB-IRIS patients were shown to have a shorter duration between TB treatment and cART when compared to the non-IRIS controls.
